# A New Design for the Fixed Primer Regions in an Oligonucleotide Library for SELEX Aptamer Screening

**DOI:** 10.3389/fchem.2020.00475

**Published:** 2020-06-05

**Authors:** Bin Wang

**Affiliations:** Department of Chemistry, Marshall University, Huntington, WV, United States

**Keywords:** systematic evolution of ligands by exponential enrichment (SELEX), aptamer screening, oligonucleotide library, fixed primer regions, i-motif

## Abstract

Single-stranded DNA or RNA oligonucleotides, often called aptamers, can bind to a molecular target with both high affinity and selectivity due to their distinct three-dimensional structures. A technique called systematic evolution of ligands by exponential enrichment (SELEX) is used to screen aptamers from a random DNA or RNA pool, or library. The traditionally-designed oligonucleotides in libraries contain a randomized central region along with a fixed primer region at each end for amplifying target-bound central sequences. The single-stranded forward and reverse primer sequences may interfere with target-binding to the central region, resulting in a partial or complete loss of high-affinity aptamers during the SELEX process. To address this issue, researchers have modified the traditional oligonucleotide libraries and developed new types of oligonucleotide libraries; however, these approaches come with various limitations. The author proposes a new design that uses a conformation-changeable sequence as primers, which may open a new avenue for developing an optimized aptamer sequence with both high affinity for, and selective binding to, a particular target via SELEX.

Aptamers are single-stranded DNA or RNA oligonucleotides that bind to a molecular target with both high affinity and selectivity due to their distinct three-dimensional structures. Target-specific aptamers are mostly screened from a random DNA or RNA pool using a technique called systematic evolution of ligands by exponential enrichment (SELEX), which involves repetitive rounds of (1) partitioning target-bound aptamers from those not bound to the target, and (2) amplifying target-bound aptamers by the polymerase chain reaction (PCR).

Since its emergence in the 1990s, the conventional SELEX technique has undergone substantial modifications and improvements to allow the aptamer screening process to be both simpler and more efficient (Berezovski and Krylov, 2002; Mendonsa and Bowser, 2004; Berezovski et al., 2005). For example, coupling the high resolving power of capillary electrophoresis (CE) to traditional SELEX produced a substantial improvement (Mendonsa and Bowser, 2005; Mosing et al., 2005). The CE-SELEX process occurs in free solution (i.e., no need for a solid support medium), isolates high-affinity aptamers in just two to four rounds, and does not require negative screening. This flexible and high-resolution approach has allowed the successful screening of aptamers capable of targeting both macromolecules such as proteins, and small compounds such as porphyrin (Yang and Bowser, 2013; Dong et al., 2015).

The traditionally-designed DNA or RNA oligonucleotides in libraries contain a randomized central region of 30–60 nucleotides, and a fixed primer region of ~20 nucleotides at each end. The target molecule tightly binds to the central region of some of the library's fragments. However, the single-stranded forward and reverse primer sequences, which are used to amplify target-bound central sequences via PCR, may also interact with/anneal to complementary sequences in the central region, thus interfering with target-binding and resulting in a partial or complete loss of high-affinity aptamers. Furthermore, the primer sequences can participate in non-specific selection by binding to the target themselves, thus leading to false-positive results since the screened aptamers (i.e., the central sequences) do not actually bind to the target molecule (Pan and Clawson, 2009). The fixed primer regions of the oligonucleotides in libraries thus play a central role in limiting the efficiency of SELEX aptamer screening.

To address this issue, researchers have attempted to modify the traditional oligonucleotide libraries or develop new types of oligonucleotide libraries. For example, Haynes' group has modified the standard nucleic acid library by introducing nucleotides that are complementary to the fixed primer regions of the library's fragments, thus eliminating the possibility of the single-stranded primer regions becoming involved in forming any aptamer-like structures (Ouellet et al., 2014). This strategy, however, has a drawback: Since some protein families (e.g., transcription factors) preferentially bind to duplexed nucleic acids, the now double-stranded primer regions in Haynes' design create binding sites for those proteins.

Other strategies include replacing the fixed primer regions with different sequences after each round of screening or after a certain number of rounds (Shtatland et al., 2000), completely removing the fixed regions and then regenerating these regions after each round of screening (Wen and Gray, 2004; Jarosch et al., 2006; Pan et al., 2010; Lai and DeStefano, 2011), or minimizing the length of the fixed primer regions (Pan et al., 2008). These approaches have drawbacks as well, such as the involvement of many steps of cleavage and re-ligation, which is time-consuming and labor-intensive, and often causes a significant loss of the library material; or the apparent decrease of the PCR efficiency due to the reduced length of the forward and reverse primer sequences, which causes the loss of the screened aptamers.

To the author's knowledge, no study has investigated the use of highly structured, self-folded forward and reverse primers to replace the standard single-stranded primers. This may be because the self-folded primer regions are aptamer-like structures themselves, and are more likely to bind to target molecules than single-stranded primer sequences. However, the use of primers that change their conformation under different conditions may actually lead to a higher selection of the target molecule. In this study, the author proposes this new idea for primer design to be used during SELEX aptamer screening.

First, as illustrated in Figure 1, the binding buffer is adjusted to Condition 1, so that the forward and reverse primer regions can self-fold into a highly structured conformation. Previous studies have demonstrated that, unlike in the conventional SELEX method, the target may bind to the randomized central region of the oligonucleotide library and/or to the folded primer regions. This is because there is little or no sequence homology among the aptamers identified using the CE-SELEX approach (Mosing et al., 2005; Yang and Bowser, 2013). Next, the target-bound fraction is separated from the unbound library, which is achieved on a CE system; the bound fraction is then collected into a vial. Since the bound fraction includes unwanted aptamers (i.e., those in which only the primer regions bind to the target), the buffer in the collecting vial is then adjusted to Condition 2, so that the self-folded primer structures unwind and the bound target is dislodged. Aptamers that use their central regions to bind to the target are not affected under Condition 2 if their central motifs do not undergo a conformation change that would release the target. The third step is to re-inject the collected solution onto the CE system, and further separate the target-bound aptamers from the unbound ones under Condition 2. This final screening produces only aptamers that have the target bound to their central regions.

**Figure 1:**
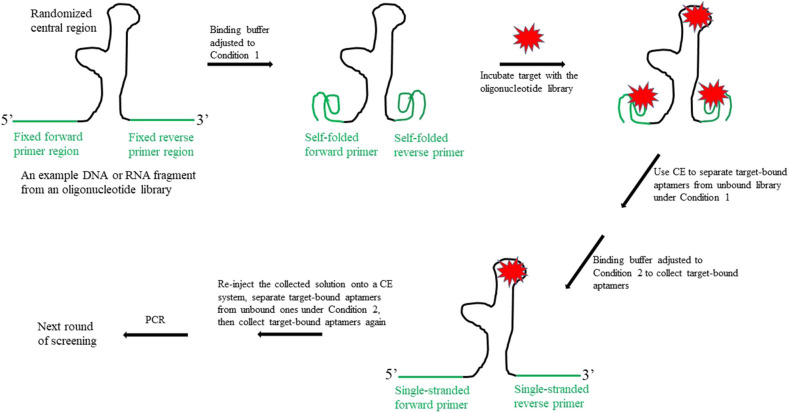
Schematic illustration of the new design for the fixed primer regions in an oligonucleotide library to be used for SELEX aptamer screening.

This new primer design requires the use of conformation-changeable DNA or RNA sequences as primers. A potential choice for such a primer is an intercalated motif (i-motif). The i-motif is a four-stranded quadruplex structure formed by cytosine-rich DNA and RNA sequences. A cytosine nucleobase may protonate at its N3 position under slightly acidic conditions, thus allowing the formation of a C^+^:C base pair between one protonated and one unprotonated cytosine via three hydrogen bonds. The C^+^:C base pairs hold two parallel cytosine tracts together, which intercalate in an antiparallel-oriented C^+^:C duplex to form a four-stranded i-motif (Abou Assi et al., 2018). The stability of an i-motif depends on factors such as the pH and ionic strength of the solution, the DNA or RNA sequence, the length of the cytosine tract, the length and nucleobase composition in the loop regions (i.e., the nucleotides that connect the cytosine tracts in an i-motif), and the temperature. By adjusting the ionic compositions and the ionic strength of both the binding and separation buffers used in the SELEX process, modifying the length of the four cytosine tracts in an i-motif, and altering the length and/or the nucleobase composition in the loop region, an i-motif can stably fold at physiological pH (~7.2) (Fujii and Sugimoto, 2015; Gurung et al., 2015; Reilly et al., 2015; Benabou et al., 2016; Wright et al., 2017). The folded i-motif structure is disrupted and becomes single-stranded when exposed to a higher pH (e.g., ~8.0). The use of aptamers designed to include i-motifs with somewhat different sequences as the forward and reverse primers in an oligonucleotide library allows the differentiation of the 5′ and 3′ ends of the oligonucleotides for PCR purposes. With this design, the target-binding process first occurs at Condition 1 (i.e., pH ~7.2); the self-folded i-motif primers do not interrupt the binding of a target molecule to the central region of the library's fragments. The bound aptamers are then separated from the unbound library fragments by CE and collected into a vial containing a buffer at approximately pH 8.0 (Condition 2). The high pH causes any target molecules that bound to the i-motif primer regions in the previous step to be dislodged due to the pH-mediated conformational change of the i-motif. The collected solution is then re-injected onto the CE system, and the bound aptamers are separated from the unbound ones at Condition 2 (i.e., pH ~8.0). Phosphate buffer is a candidate for the CE running/separation buffer, as it can be adjusted to both pH 7.2 (Condition 1) and 8.0 (Condition 2), without losing essential buffering characteristics; an uncoated fused-silica capillary is sufficient to run the entire experiment under both pH conditions. The aptamers screened using this new approach will have a high affinity to the target molecule at both physiological pH and a higher pH (~8.0).

Studies have demonstrated that C-rich i-motifs and G-rich G-quadruplexes can co-exist if one or both are stably folded (Phan and Mergny, 2002; Kumar et al., 2008; Bucek et al., 2009). In the new design described above, the self-folded i-motif primers that exist under Condition 1 will not interact with any G-rich sequences in the randomized central region since the base-pairing energy for the C^+^:C pair in an i-motif (169.7 kJ/mol) is much higher than that of canonical Watson-Crick GC pair (96.6 kJ/mol) (Yang and Rodgers, 2014). Under Condition 2, the i-motif primer regions become single-stranded; however, the likelihood that they would interrupt the identification of G-quadruplex aptamers in the central region is low because G-quadruplex motifs can stably fold under higher pH (~8.0). In addition, the target of interest has already bound to the central region of certain library fragments under Condition 1. If, under Condition 2, some of these potential aptamers lose their affinity for the target due to pH-induced conformational changes or interaction with now single-stranded primer sequences, our goal of only screening aptamers with high affinity to the target molecule at both physiological pH and a higher pH (~8.0) is achieved.

In addition to the i-motif that responds to a change in pH, other potential primers include DNA or RNA motifs that change their conformation in response to changes in temperature, or to changes in the composition of the binding buffer, such as a change in the concentration of either Mg^2+^ or a monovalent ion. The author asserts that this new type of conformation-changeable primer may render more flexibility for developing an optimized aptamer sequence with both a high affinity for and selective binding to a particular target under different conditions, and could thus be the future direction for the construction of libraries developed for SELEX aptamer screening.

## Data Availability Statement

All datasets generated for this study are included in the article/supplementary material.

## Author Contributions

The author confirms being the sole contributor of this work and has approved it for publication.

## Conflict of Interest

The author declares that the research was conducted in the absence of any commercial or financial relationships that could be construed as a potential conflict of interest.
